# Metformin Administration During Early Postnatal Life Rescues Autistic-Like Behaviors in the BTBR T+ Itpr3tf/J Mouse Model of Autism

**DOI:** 10.3389/fnbeh.2018.00290

**Published:** 2018-11-29

**Authors:** Lian Wang, Yulong Cai, Xiaotang Fan

**Affiliations:** Department of Developmental Neuropsychology, School of Psychology, Third Military Medical University, Chongqing, China

**Keywords:** autism, sociability, BTBR mouse, metformin, repetitive behavior

## Abstract

Autism spectrum disorder (ASD) is a neurodevelopmental disability characterized by impaired social interactions, stereotypical repetitive behavior and restricted interests. Although the global incidence of ASD has increased over time, the etiology of ASD is poorly understood, and there is no effective pharmacological intervention for treating ASD. Recent studies have suggested that metformin has the potential to treat ASD. Thus, in this study, we assessed the therapeutic effects of early metformin treatment in a BTBR T+ Itpr3tf/J (BTBR) mouse model of ASD. We observed that early metformin administration significantly reversed social approach deficits, attenuated repetitive grooming and reduced marble burying in BTBR mice. Metformin did not change the general locomotor activity or anxiety-like behavior in both BTBR and C57BL/6J (B6) mice. Our findings suggest that early metformin treatment may have beneficial effects on ameliorating behavioral deficits in ASD.

## Introduction

Autism spectrum disorders (ASDs) are a cluster of neurodevelopmental disabilities characterized by defective social interactions, impaired communication and restricted and repetitive behaviors (Leekam et al., 2011; Fakhoury, 2015). About 1% of children are affected by ASD all over the world, and the prevalence is allegedly increasing with even more unreported and undiagnosed ASD cases (Huguet et al., 2016). Despite its significance, there are still no validated pharmacological or behavioral treatments to alleviate ASD symptoms (Chahrour et al., 2016; Lacivita et al., 2017). Therefore, it is urgent for efficacious treatments to be developed to prevent and treat ASD.

Metformin, a biguanide drug, is widely utilized in the therapy of diabetes mellitus. It was demonstrated that metformin crosses the blood brain barrier and it was amply demonstrated to not only prevent diabetes-induced memory deficits in rats (Mousavi et al., 2015), but also modulate cognitive functional abnormalities in several neuropsychiatric disorders (Hsu et al., 2011; Dy et al., 2018). In depressed patients with diabetes mellitus, chronic treatment with metformin has antidepressant behavioral effects through improvements in cognitive performance (Guo et al., 2014). Metformin treatment rescued spatial memory impairment and neuronal death in the hippocampus, and promoted hippocampal neurogenesis in Alzheimer’s disease mouse model (Ou et al., 2018). One clinical study conducted by Dy et al. (2018) has shown that metformin treatment could cause consistent alleviation in social responsiveness, social avoidance, irritability and hyperactivity in individuals with fragile X syndrome (FXS), the leading inherited cause of intellectual disability and ASD, without significant side-effects. A recent study has also shown that metformin corrected social deficits, reversed increased self-grooming and reduced the incidence of seizures in the adult FXS mouse model (Gantois et al., 2017). These data suggest that metformin may have the potential to counteract the cognitive and behavioral deficits shown in ASD models.

It has been inferred that early intervention is important to prevent and correct the pathological phenotypes of neurodevelopmental disorders, thereby reversing behavioral and functional deficits in adults (Cioni et al., 2016). Treatment with Metformin significantly improved deficits of locomotor activity and cognition in rats subjected to perinatal hypoxia-ischemia (Qi et al., 2017). Another study indicated that metformin administration during the neonatal period could alleviate a contextual fear memory deficit in a fetal alcohol spectrum disorder model (Tunc-Ozcan et al., 2018). One preclinical study reported that early social enrichment could rescue adult behavioral and spine morphology abnormalities shown in a FXS mouse model (Oddi et al., 2015). The study conducted by Vuillermot et al. (2017) revealed that vitamin D treatment during pregnancy could prevent autism-relevant behavioral abnormalities in an ASD mouse model. However, the role of early metformin treatment in the prevention-correction remains unclear.

The BTBR T+ Itpr3tf/J (BTBR) inbred mouse strain, a well-characterized animal model, displays several ASD-related behaviors. Similar to the clinical symptoms of ASD, BTBR mice display low sociability and increased repetitive behaviors, like self-grooming and marble burying (McFarlane et al., 2008; Silverman et al., 2010; Amodeo et al., 2016). In the present experiment, we examined the effect of early metformin treatment on social and repetitive behavioral deficits in the BTBR mice. The effect of metformin on social and repetitive behaviors was also examined in the control strain, C57BL/6J (B6), which displays normal sociability and a low level of repetitive behavior. This preclinical study shows that metformin has the potential of being a pharmacological target for ameliorating the core symptoms of ASD.

## Materials and Methods

### Animals

Mice were bred according to the Third Military Medical University guidelines and 2–4 mice were grouped per cage under a 12:12 h light:dark cycle. All mice were maintained in a standard animal facility with water and food *ad libitum*. BTBR mice breeding pairs were purchased from the Model Animal Research Center of Nanjing University (Nanjing, China). The B6 mice breeding pairs were obtained from the Third Military Medical University. Male pups used in our studies were all bred in the same facility of the Third Military Medical University. This study was carried out in accordance with the recommendations of Laboratory Animal Welfare and Ethics Committee of the Third Military Medical University. The protocol was approved by the Laboratory Animal Welfare and Ethics Committee of the Third Military Medical University. All efforts were made to minimize the suffering of animals and the number of animals used.

### Drug Treatment

**Figure 1:**
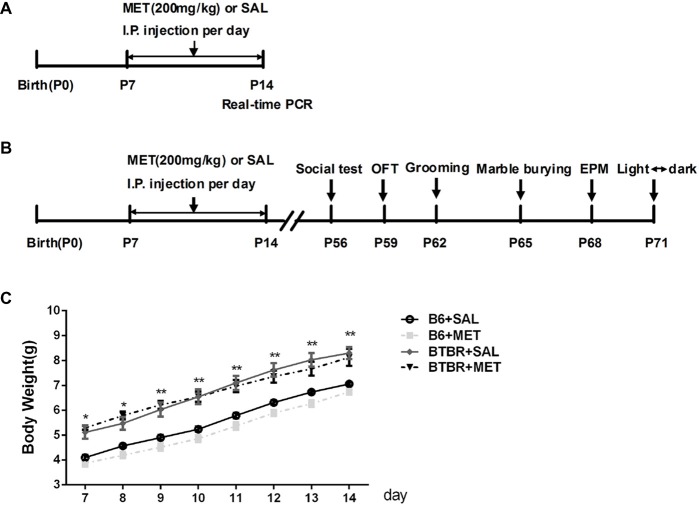
Schematic diagram of the experimental procedures. **(A)** Neonatal male mice (B6 or BTBR) were injected with metformin (MET; 200 mg/kg) or saline (SAL) from P7 to P14 once per day. **(B)** The behavior tests were conducted at the age of 8 weeks. **(C)** The effects of MET on the average body weight of mouse pups. OFT, Open Field Test; EPM, Elevated Plus-Maze. *p < 0.05, **p < 0.01.

To avoid the litter effects, we randomly chose a maximum of two male pups per litter for each experimental group. B6 groups were composed of offspring from 10 different litters and BTBR from 9 litters. Postnatal day 0 (P0) refers specifically to the day of birth. Fourteen male pups in each group were intraperitoneally (i.p.) injected with metformin (200 mg/kg), a dose previously used in animal studies (Gantois et al., 2017; Tunc-Ozcan et al., 2018) or the vehicle (same volume of sterile saline solution) from P7 to P14 once per day (Figure 1A). The treatment groups included the following: B6+Saline (SAL), B6+metformin (MET), BTBR+SAL, BTBR+MET.

### Behavioral Tests

Behavioral tests of BTBR and of the control strain B6 were conducted at the age of 8 weeks during daytime of the circadian cycle (each group contains 10 mice). The mice were subjected to a comprehensive battery of behavioral tests; the timeline for behavioral testing is shown in Figure 1B. Mice were acclimated to the behavior laboratory for at least 30 min prior to each test.

### Three-Chambered Social Approach

The testing procedures were evaluated in a rectangular apparatus (40 cm × 60 cm × 22 cm, divided into three equal parts) as previously described (Yang et al., 2011). In brief, the test had a 10-min habituation phase and a 10-min testing phases (object vs. stranger mouse). During the habitation period, a test mouse was introduced and allowed to freely explore the whole arena. Then, the subject animal explored all three chambers for 10 min, where they were given the choice to interact with a novel caged mouse (S) on one side of the chamber, or stay on the other side of the chamber with a novel object (O). The chambers were cleaned using 70% ethanol and water between each test. The amount of time spent in each chamber sniffing, and the number of entries into each chamber were analyzed utilizing Ethovision XT 11.5. The preference index was calculated, which represents a numerical alteration numerical between the time in the chambers (stranger mouse vs. object) divided by the total time spent in both chambers.

### Open Field Test

General open field locomotion was assessed in a photocell-equipped automated open field arena (40 cm × 40 cm × 30 cm). The animals were placed in the center of the apparatus and allowed to move around freely for 30 min. The movements of the mice were recorded for 30 min using a video camera secured to the top of the apparatus and analyzed using Ethovision XT 11.5. The test apparatus was cleaned with 70% ethanol between each subjects test session. The testing room was illuminated at ~200 lux (Silverman et al., 2015).

### Self-Grooming

The mice were introduced gently into a clean standard mouse cage with a video camera placed 15 cm away from the cage. Twenty minutes were recorded for the manual scoring of repetitive self-grooming during the second 10 min of the testing session, illuminated at 40 lux. The total amount of time spent grooming, number of grooming bouts and the percentage of incorrect grooming transitions were analyzed by an observer that was blind to the experimental design. The grooming behavioral microstructure was analyzed using a grooming analysis algorithm. A grooming bout consists of the following grooming steps: no grooming (0), paw (1), face (2), body (3), leg (4), tail/genital (5). A complete bout includes an in-order grooming from 0 to 5, without any longer than 6’s interruption and no other behavior during the bout. We defined a sequentially transition move in the correct order, as 0–1, 1–2, 2–3. The incorrect transitions of disordered grooming movements, are defined as 1–3, 4–1 (Kalueff et al., 2007; Pearson et al., 2011). The percentage of incorrect transitions was calculated as incorrect transitions divided by total number of transitions. The cages were cleaned with 70% ethanol between each subjects test session (Silverman et al., 2015).

### Marble Burying

The marble burying was measured by previously described methods (Gould et al., 2011). In brief, empty and clean cages were filled with bedding up to 5 cm in depth, and 20 black glass marbles were arranged in a 4 × 5 grid throughout the bedding. The mice were then moved to the cages and allowed to freely explore for 30 min, deprived of food and liquids. Afterwards, the number of marbles buried was counted. Marble “burying” was defined as two-thirds or more covered by bedding.

### Elevated Plus-Maze

The Elevated Plus-Maze (EPM) was structured with two open arms and two closed arms (30 cm × 6 cm ×15 cm) extending from a central area (6 cm × 6 cm). Initially, the mice were placed in the center area facing an open arm. During the 10-min session, the ratio of time spent in the open arms divided by the total time spent in all arms and the total number of entries were analyzed (Cai et al., 2017). The maze was thoroughly cleaned using 70% ethanol and water after each test session.

### Light-Dark Transitions

The light-dark exploration test was used to evaluate the anxiety levels of the mice according to previously described procedures (Flannery et al., 2015). Mice were placed into the “light” side (~400 lx) and allowed to move freely for 10 min. Time spent in the dark side and the total number of transitions were automatically recorded by Noldus Observer software (Ethovision XT 11.5). The illumination of the room was ~400 lux.

### Real-Time PCR

Mice were sacrificed 2 h after the last injection of metformin at P14. The isolated cerebral cortex of the mice were used for the Real-time PCR (each group contains four mice). The total RNA was extracted using a RNeasy kit (CWBIO, Cat.CW05815, China) according to the manufacturer’s instructions. A spectrophotometric instrument (NanoDrop) was used to quantify the RNA concentration. The purified total RNA (approximately 1–2 μg per 20 μl reaction) was followed by a reverse transcription. The SYBR Green qPCR Mix (Takara) was used for Real-time PCR analysis. The specific primer sequences for mTOR, S6K, NF-κB and GAPDH are listed as follows: mouse mTOR: forward: 5′-CAGTTCGCCAGTGGACTGAAG-3′, reverse: 5′-GCTGGTCATAGAAGCGA G TAGAC-3′; mouse S6K, forward: 5′-GGGGCTATGGAAAGGTTTTTCA-3′ reverse: 5′-CGTGTCCTTAGCATTCCTCACT-3′; mouse NF-κB, forward: 5′-ATG GCAGACGATGATCCCTAC-3′ reverse: 5′-CGGAATCGAAATCCCCTCTGTT-3′; mouse GAPDH forward primer: 5-AGGTCGGTGTGAACGGATTTG-3 and reverse primer: 5-TGTAGACCATGTAGTTGAGGTCA-3. The relative expression levels were normalized to GAPDH and calculated using the 2^−ΔΔC(t)^ method.

### Western Blotting

The entire cortex was harvested and homogenized in an ice-cold RIPA lysis buffer (Beyotime, Shanghai, China) 2 h after the last injection of metformin at P14. The concentration was measured by the Bicinchoninic Acid Kit (Beyotime, Shanghai, China). The samples were separated on SDS-PAGE gel (120 min 80 V) and then transferred to a PVDF membrane (90 min at 210 mA). After being blocked with 5% fat-free milk for 3 h at RT, membranes were incubated with primary antibodies at 4°C overnight (rabbit anti-mTOR, 1:1,000, Cell Signaling; mouse anti-β-actin, 1:1,000, Cell CWBIO, Beijing, China). The bands were incubated with secondary antibodies (mouse IgG, 1:2,000, Santa Cruz Biotechnology; rabbit IgG, 1:2,000, Santa Cruz Biotechnology) for 2 h at RT and visualized by the bioimaging system (Quantity One 4.0; Bio-Rad Laboratories, Hercules, CA, USA). Three mice per group and at least three independent experiments were used for the analysis.

### Statistical Analyses

The data were presented as the mean ± SEM. Two-way analysis of variance (ANOVA) was used to evaluate results of self-grooming, marble burying, EPM, light-dark transitions and real-time PCR. Open field locomotion and body weights were analyzed with repeated measures ANOVA. Two-way ANOVA, repeated measures ANOVA and paired *t*-test were performed in a three-chambered social approach experiment, as previously described (Scattoni et al., 2008; Silverman et al., 2015). Significant effects were evaluated with the least significant difference (LSD) *post hoc* test. For all comparisons, the statistical significance was set at *p* < 0.05.

## Results

### Effects of Metformin on Average Body Weight

Body weights were measured from P7 to P14 during the treatment. An apparent effect was detected in both groups (Figure 1C, *F*_(3,36)_ = 20.906, *p* < 0.01) and time factors (Figure 1C, *F*_(7,36)_ = 557.009, *p* < 0.01), but the effect of interaction between factors (Figure 1C, *F*_(21, 36)_ = 1.697, *p* > 0.05) was not obvious. Consistent with previous research (Scattoni et al., 2008), the body weight of BTBR pups, treated with saline, was higher than B6 pups treated with saline from postnatal days 7 to 14 (Figure 1C, *p* < 0.01). However, metformin administration did not affect the body weight in B6 and BTBR mice.

### Deficits in the 3-Chambered Social Approach Task in BTBR Mice Can Be Rescued by Early Metformin Treatment

**Figure 2:**
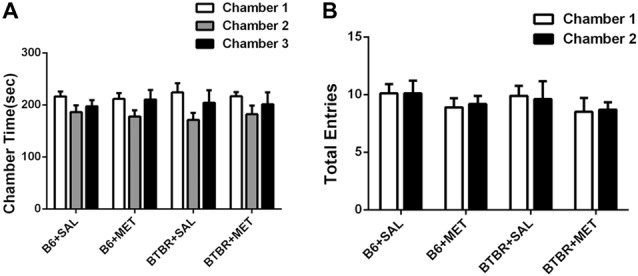
The exploratory locomotion in the three-chambered social test did not significantly affected by early metformin treatment in B6 and BTBR mice during the habituation period. **(A)** There were no differences in the chamber time in four groups during the habituation phase before the beginning of the sociability test. **(B)** The number of entries into the side chambers was similar between the four groups, indicating that metformin did not affect general exploratory activity during the test. Data are shown as the mean ± SEM in all figures. *n* = 10.

The three-chambered social test was conducted to qualitatively and quantitatively analyze social approach behaviors in rodent models of disorders, such as autism (Yang et al., 2011). During the 10-min habitation session, the B6 (Figure 2A, B6 saline: *F*_(2,27)_ = 1.801, *P* > 0.05; B6 metformin: *F*_(2,27)_ = 1.810, *P* > 0.05) and BTBR mice (Figure 2A, BTBR saline: *F*_(2,27)_ = 2.035, *P* > 0.05; BTBR metformin: *F*_(2,27)_ = 1.028, *P* > 0.05) showed no particular chamber preference in the three-chambered social task with drug treatment across the habituation. In addition, no significant difference was found in the number of total entries in B6 (Figure 2B, B6 saline: *t*_(9)_ = 0.000, *P* > 0.05; B6 metformin: *t*_(9)_ = 0.300, *P* > 0.05) and BTBR mice (Figure 2B, BTBR saline: *F*_(1, 9)_ = 0.205, *P* > 0.05; BTBR metformin: *F*_(1, 9)_ = 0.161, *P* > 0.05). This result suggests that early metformin treatment did not directly affect the exploratory activity during the habituation session preceding the social approach test in BTBR and B6 mice.

**Figure 3:**
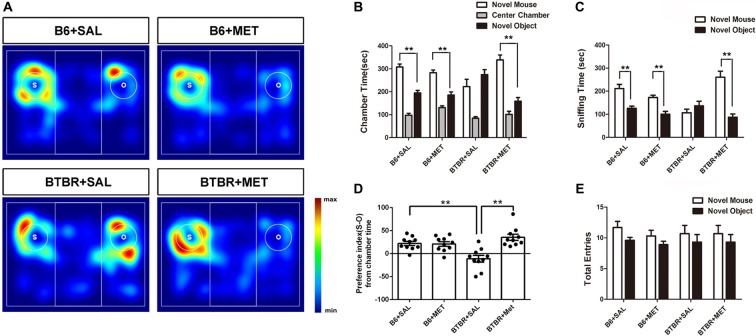
Early metformin treatment reversed social approach deficits in the BTBR mouse model of autism. **(A)** The heat maps show the total time and location in BTBR and B6 mice during the sociability chamber test. “O” and “S” represent object and mouse, respectively. Warmer colors (red) represent longer stay. **(B)** BTBR mice show that spending similar time staying with a novel object compared to a novel mouse. Early metformin treatment can reverse the social deficit by increasing the time interaction with the novel mouse. **(C)** BTBR mice spend about equal sniffing time with a novel object compared to control mice. Early metformin treated mice increased the time sniffing with the mouse. **(D)** Compared to control mice, BTBR mice showed a lower preference index (S-O/total), and early metformin treatment can improve sociability for increasing the preference index. **(E)** There was no significant difference in total entries observed in all groups. Data are presented as the means ± SEM. *n* = 10. **P < 0.01.

Sociability is defined as a preference for spending more time with the novel mouse than with the novel object. In the present study, B6 mice spent significantly more time in the chamber containing the novel mouse as compared to the time spent in the chamber with the nonsocial novel object, in both the saline- (Figures 3A,B, *t*_(9)_ = −4.869, *P* = 0.001) and the metformin-treated group (Figures 3A,B, *t*_(9)_ = −4.108, *P* = 0.003). Saline treated BTBR mice showed neither preference for the novel mouse chamber nor the novel object chamber (Figures 3A,B, *t*_(9)_ = −1.434, *P* > 0.05). However, metformin treatment did rescue the sociability deficit of BTBR mice as conformed by the increased social preference for the novel mouse chamber (Figures 3A,B, *t*_(9)_ = −5.157, *P* = 0.001), displaying restoration of the characteristic social behavioral abnormalities.

We also measured the total time spent sniffing the novel caged mouse and the novel object in each group (Figures 3A,C). B6 mice treated with saline or metformin during early postnatal life spent significantly more time sniffing the novel mouse than sniffing the novel object (Figure 3C, *P* < 0.01). BTBR mice treated with saline showed no significant difference in the total time spent sniffing the novel object than the novel mouse (Figure 3C, *t*_(9)_ = −1.072, *P* > 0.05). However, BTBR mice treated with metformin did spend significant more time sniffing the novel mouse than time sniffing the novel object (Figure 3C, *t*_(9)_ = −5.038, *P* = 0.001), which indicated improved social sniffing and more time engaging with a social stimulus.

To further quantify changes in sociability following metformin treatment during early postnatal life, the social preference index was assessed across the four groups. A two-way ANOVA indicated a main effect of genotype across the social preference index (Figure 3D, *F*_(1,36)_ = 2.456, *P* < 0.01), B6 mice displaying a high social preference index compared with BTBR mice regardless of treatment. In addition, there was a significant drug effect in BTBR mice (Figure 3D, *F*_(1,36)_ = 14.711, *P* < 0.01), as well as a drug by genotype interaction (Figure 3D, *F*_(1,36)_ = 16.255, *P* < 0.01), with metformin treated BTBR mice displaying a significantly increased social preference index compared to early saline treated BTBR mice (Figure 3D, *P* < 0.01). These differences in the social preference index were not detected between treatment groups in B6 mice. Together, these data indicate that metformin administered to BTBR mice during early postnatal life improved social approach behaviors.

In addition, we showed that entries into the side chambers were not affected by metformin administration in B6 or BTBR mice (Figure 3E), indicating that the exploratory locomotion of the mice were not changed during the social approach task.

### Early Metformin Treatment Reduced Repetitive Self-Grooming and Marble Burying Behaviors in BTBR Mice

**Figure 4:**
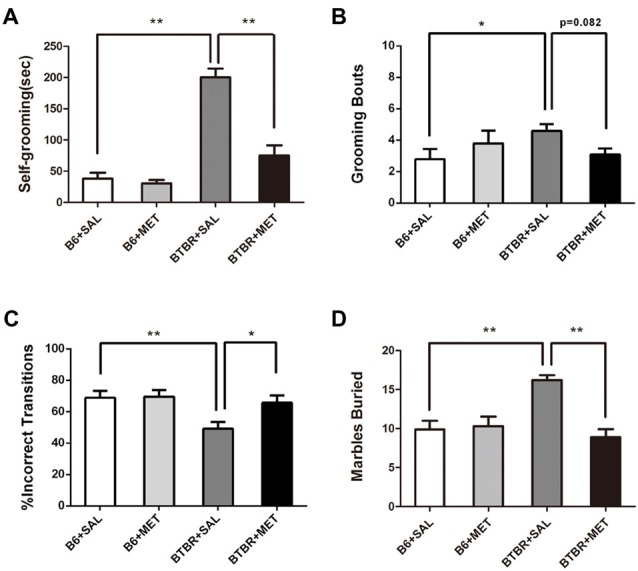
Early metformin treatment rescued the repetitive behaviors in BTBR mice.** (A)** BTBR mice showed severe repetitive behaviors due to high levels of self-grooming compared to B6 mice, and early metformin treatment can reverse it. **(B)** Metformin treatment tended to reduce grooming bouts in BTBR mice compare with saline-treated BTBR mice, the effect approached statistical significance. **(C)** BTBR mice showed decreased proportion of incorrect transitions compared to B6 mice, and metformin treatment increased it. **(D)** BTBR mice buried more marbles compared to B6 mice, and metformin treatment can reverse it. Data are presented as the means ± SEM. *n* = 10. *P < 0.05, **P < 0.01.

It was established that BTBR mice exhibited multiple patterns of repetitive and stereotypical behaviors. We therefore further investigated the effects of early postnatal metformin treatment on the time of self-grooming and number of marble burying, as measures of stereotypical repetitive behaviors (Figure 4A). Saline-treated BTBR mice also exhibited significantly higher self-grooming behavior compared with saline-injected B6 mice. There was a significant drug effect in BTBR mice (Figure 4A, *F*_(1,36)_ = 30.115, *P* < 0.01) as well as a treatment by genotype interaction (Figure 4A, *F*_(1,36)_ = 23.096, *P* < 0.01), with early metformin treated BTBR mice displaying a significantly increased social preference index compared to early saline treated BTBR mice (Figure 4A, *P* < 0.01). An early postnatal metformin treatment did not change self-grooming behavior in B6 mice.

Saline-treated B6 mice revealed significantly lower grooming bouts compared with saline-injected BTBT mice. The two-way ANOVA analysis showed no significant effect of genotype (Figure 4B, *F*_(1,36)_ = 0.861, *P* = 0.360) or early metformin treatment (Figure 4B, *F*_(1,36)_ = 0.178, *P* = 0.676) on grooming bouts. The grooming bouts were significantly altered by drug × genotype interaction (Figure 4B, *F*_(1,36)_ = 4.447, *P* = 0.042). *Post hoc* analysis revealed that early metformin treatment decreased grooming bouts in BTBR mice compared to saline-treated BTBR mice, and this effect approached statistical significance (Figure 4B, *P* = 0.082).

The percentage of sequentially incorrect grooming transitions was significantly altered by the genotype (Figure 4C, *F*_(1,36)_ = 7.274, *P* = 0.011), with non-significant results in the drug treatment (Figure 4C, *F*_(1,36)_ = 3.974, *P* = 0.054) and drug × genotype interaction (Figure 4C, *F*_(1,36)_ = 3.199, *P* > 0.05). *Post hoc* analysis revealed that saline-treated BTBR mice exhibited a reduced percentage of sequentially incorrect grooming transitions compared to the saline-treated B6 mice (Figure 4C, *P* < 0.01). Additionally, early metformin treatment sequentially increased the percentage of sequentially incorrect grooming transitions in BTBR mice compared to saline-treated BTBR mice (Figure 4C, *P* < 0.05).

The marble burying test was also used as an evaluation of repetitive behavior (Gould et al., 2011). There was a drug effect (Figure 4D, *F*_(1,36)_ = 11.351, *P* < 0.01), genotype effect (Figure 4D, *F*_(1,36)_ = 5.724, *P* < 0.05), and drug × genotype effect (Figure 4D, *F*_(1,36)_ = 14.135, *P* < 0.01) for the marble burying test. *Post hoc* analysis revealed that saline-injected BTBR mice buried a higher number of marbles compared to saline-injected B6 mice (Figure 4D, *P* < 0.01). Metformin treated BTBR mice significantly reduced buried marbles compared to saline-injected BTBR mice (Figure 4D, *P* < 0.01). However, metformin treatment did not significantly affect the number of marbles buried by B6 mice.

### Effects of Early Metformin Treatment on Locomotion and Anxiety in BTBR Mice

**Figure 5:**
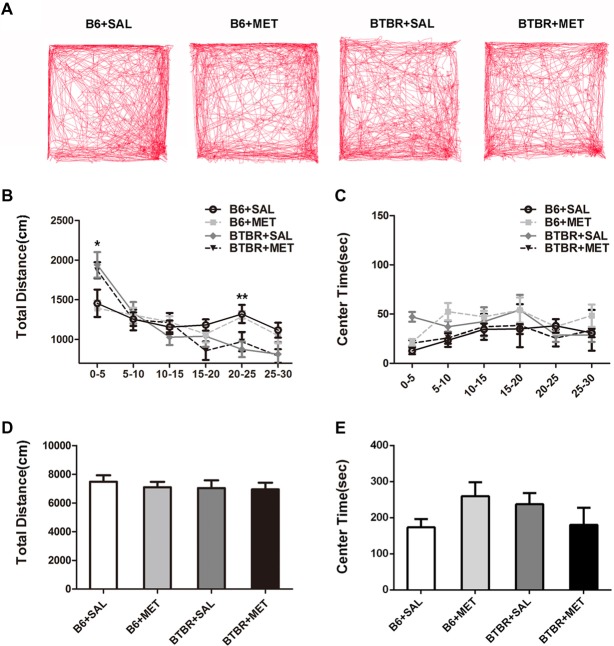
Metformin treatment did not alter exploratory locomotion in the open field in B6 or BTBR mice. **(A)** The representative traces of different group mice in open field test show a 30-min assay of distance moved and time in center were computationally split into six segments with 5 min per segment. Compared with vehicle treated group, early metformin treatment did not have effects on distance traveled **(B)** and time spend in center **(C)** in each segment. Across the 30-min test, total distance traveled **(D)** and time in center **(E)** was not affected by early metformin treatment in B6 and BTBR mice. Data are presented as the means ± SEM. *n* = 10. *p < 0.05, **p < 0.01.

Since general locomotor activity and exploratory behavior could confound the results detected in the three chambered social tasks and self-grooming, we conducted an open-field locomotion test in BTBR and B6 mice. During the 30 min testing period, the total distance in the open field decreased significantly across time (Figures 5A,B, *F*_(5,36)_ = 34.419, *P* < 0.01), indicating that mice could habituate to the novel environment normally. There was no significant time in the genotype interaction (Figure 5B, *F*_(5,36)_ = 0.258, *P* = 0.855). We found a significant main effect of the genotype during 0–5 min (Figure 5B, *F*_(1,36)_ = 12.195, *P* < 0.01) and 20–25 min (Figure 5B, *F*_(1,36)_ = 13.255, *P* < 0.01). This means BTBR groups showed increased locomotor activity during 0–5 min while showing deceased activity during 20–25 min. Higher initial open field activity of the BTBR was also reported in other previous studies (McFarlane et al., 2008; Silverman et al., 2010). The two-way ANOVA analysis showed there was no drug effect (Figure 5D, *F*_(1,36)_ = 0.258, *P* = 0.614), genotype effect (Figure 5D, *F*_(1,36)_ = 0.404, *P* = 0.529), and drug × genotype effect (Figure 5D, *F*_(1,36)_ = 0.100, *P* = 0.754) for the total distance, revealing the absence of any confounding hypo- or hyperactivity effect. Mice with higher levels of anxiety preferred spending time in the non-center regions of an open field as they became habituated to the novel environment. There was no significant change of time in the genotype interaction (Figure 5C, *F*_(5,36)_ = 0.138, *P* = 0.264), in the center time during the 30 min session. The center time was not altered by the genotype (Figure 5C, *F*_(1,36)_ = 0.044, *P* = 0.834) or metformin treatment (Figure 5C, *F*_(1,36)_ = 0.161, *P* = 0.691). Although there were no significant drug × genotype effects for the center time, a trend was observed (Figure 5E, *F*_(1,36)_ = 3.936, *P* = 0.055). This indicated that metformin treatment has no effect on general anxiety in the Open Field Test (OFT).

**Figure 6:**
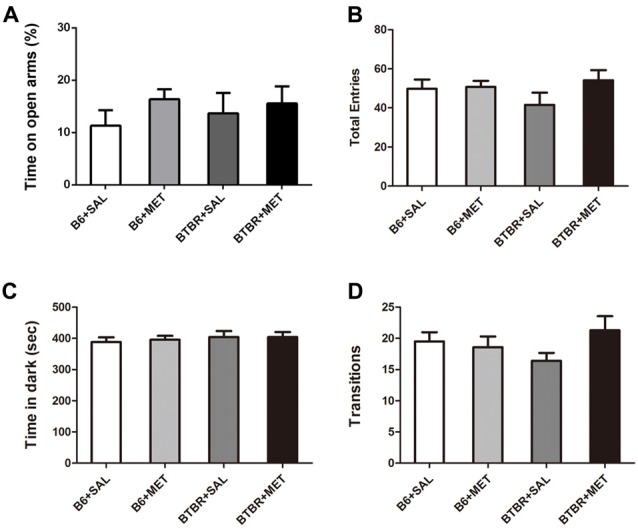
Early metformin treatment did not induce anxiety-like effects in the EPM and standard light-dark transitions. **(A)** Early metformin treatment didn’t alter the percent of time in open arms in B6 and BTBR mice. **(B)** The total entries in B6 and BTBR mice were not affected by early metformin treatment. **(C)** Early metformin treatment didn’t change time spent in dark box in B6 and BTBR mice. **(D)** Number of transitions between the light and dark compartments exhibited identical scores in all groups. Data are presented as the means ± SEM. *n* = 10.

The anxiety effects of early metformin treatment were assessed using two paradigms: an EPM test and light-dark transitions. A two-way ANOVA analyses revealed no significant effects for the genotype, drug and drug × genotype on the percent of time spent in open arms (Figure 6A, *F*_(1,36)_ = 0.067–1.253, *P* = 0.270–0.798), as well as the total number of entries (Figure 6B, *F*_(1,36)_ = 0.236–1.837, *P* = 0.184–0.630). Analysis using a light-dark box further found no significant effects for the genotype, drug and drug × genotype on the cumulative time spent in the dark chamber (Figure 6C, *F*_(1,36)_ = 0.055–0.551, *P* = 0.463–0.815), as well as for the transitions between the light and dark compartments (Figure 6D, *F*_(1,36)_ = 0.004–2.396, *P* = 0.130–0.953). These data suggest that the level of anxiety in these two paradigms was not altered in these mice. Therefore, early metformin treatment did not induce anxiety-like behavior in the OF, EPM and light–dark box tasks.

### Effects of Early Metformin Treatment on mTOR, S6K and NF-κB Expressions in the Cerebral Cortex of Mice at P14

**Figure 7:**
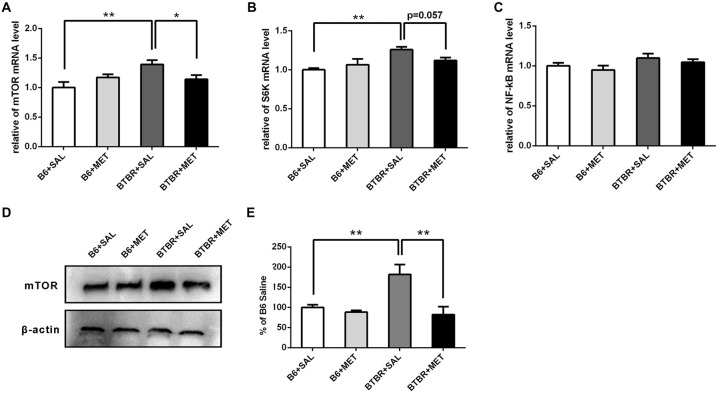
Expression of mTOR, S6K, NF-κB and the protein levels of mTOR in the cerebral cortex of B6 and BTBR mice. **(A)** BTBR mice showed an increased expression of mTOR in the cerebral cortex, and metformin treatment could decrease mTOR mRNA level in BTBR mice. **(B)** BTBR mice showed an increased expression of S6K in the cerebral cortex, and a decreased trend of S6K level was seen after metformin treatment. **(C)** The expression of NF-κB was not affected by the metformin treatment. Data are presented as the means ± SEM. *n* = 4. **P < 0.01, *P < 0.05. **(D)** Representative western blot image for mTOR protein expression. **(E)** Quantitative data of mTOR protein expression in the cortex of the mice. Data are expressed as the means ± SEM (*n* = 3 in each group; **P < 0.01).

We further noticed that the mRNA levels of target rapamycin (mTOR) and the ribosomal protein p70S6 kinase1 (S6K), a downstream target of mTOR in BTBR the cerebral cortex, were higher than in the B6 cerebral cortex. After administration of metformin, relative levels of mTOR mRNA were significantly decreased in BTBR mice (Figure 7A, main effect of drug: *F*_(1,12)_ = 0.277, *P* = 0.609; main effect of the genotype: *F*_(1,12)_ = 5.802, *P* < 0.05; drug × genotype effect *F*_(1,12)_ = 5.802, *P* < 0.05). Although the difference was not significant (Figure 7B, *P* = 0.057), a decreasing trend of S6K mRNA levels was observed after metformin treatment in BTBR mice (Figure 7B, main effect of drug: *F*_(1,12)_ = 0.629, *P* = 0.443; main effect of genotype: *F*_(1,12)_ = 11.435, *P* < 0.01; drug × genotype effect *F*_(1,12)_ = 4.780, *P* < 0.05). However, metformin did not alter the expression of mTOR and S6K in B6 mice. We also found that there were no significant differences in the expression of NF-κB between B6 and BTBR mice (Figure 7C, effects for genotype, drug and drug × genotype: *F*_(1,12)_ = 0.000–4.267, *P* = 0.061–0.983). Consistent with the result of mRNA levels, metformin administration significantly reduced levels of mTOR protein (Figures 7D,E, two-way ANOVA: main effect of drug: *F*_(1,8)_ = 11.703, *P* < 0.01; main effect of genotype: *F*_(1,8)_ = 5.406, *P* < 0.05; drug × genotype effect *F*_(1,8)_ = 7.262, *P* < 0.05) in BTBR mice, without affecting the levels in B6 cortex. This finding indicates that metformin can reverse the dysregulation of mTOR pathway in the BTBR mice.

## Discussion

The major results of this study reveal that neonatal metformin treatments, administered from P7 to P14, can improve social interaction and reduce some repetitive behaviors in BTBR mice. Our study suggests that metformin treatment, during neonatal development, might play a role in preventing some of the behavioral abnormalities associated with ASD and that the BTBR mouse model can be a promising model for the preclinical screening of suitable drugs.

The BTBR mice and the control strain B6 mice were used to investigate the efficacy of pharmacological agents on the modification of autism-related phenotypes in the BTBR model, as a potential preclinical translational strategy to screen valuable therapies for ASDs (Kazdoba et al., 2016). In the 3-chambered test for social approach, BTBR mice treated with metformin during neonatal development showed a strong preference for the novel mouse compared to the object, revealed by the time spent in the chamber and sniffing. While the exploratory activity and motor abilities of mice could affect their social behaviors, we further evaluated the effects of metformin on exploratory locomotion. The chamber entries made by mice with early metformin treatment were not significantly different than the chamber entries made by mice with early saline treatment, neither during the sociability test period nor during the habituation period that preceded the social approach task, indicating no confounding effects of early metformin treatment on locomotion. We also conducted a standard 30 min OFT to assess the general exploratory locomotion of mice. The total distance traveled by mice receiving early metformin treatment was not significantly different than that of mice with vehicle treatment, neither in B6 nor BTBR mice. This result indicated that the improvement of social impairments by early metformin treatment was typical in BTBR mice.

Self-grooming behavior represents a highly stereotypical pattern of movements which may model obsessive, compulsive-like behaviors when elevated in rodents (Silverman et al., 2015). The results of self-grooming were similar to previous studies, which demonstrated that BTBR mice spent significantly more time grooming themselves than the control B6 mice. Early metformin treatment could reduce the time spent self-grooming in the BTBR mice, but not in the B6 mice. In this study, we also analyzed the percentage of incorrect grooming transitions, which could evaluate rigidity displayed by the mice (Kalueff et al., 2007). The BTBR mice groomed with a lower percentage of sequentially incorrect grooming transitions, suggesting more rigidity than the control C57BL/6J mice. Early metformin treatment decreased rigidity in the BTBR mice, defined as an increased percentage of sequentially correct grooming transitions. The marble burying test was developed as a measure of repetitive behavior (Gould et al., 2011). Remarkably, early metformin treatment reduced the number of buried marbles by BTBR mice. Since locomotion activity of either strain during the OFT was unchanged by metformin administration, the data indicated that the treatment of metformin during early development could reduce the presentation of certain repetitive behaviors.

Metformin increases anxiolytic behavior in either a diabetic rat model followed by focal ischemia, or in rats with cerebral ischemic injury (Sarkaki et al., 2015). Growing evidence has indicated that BTBR mice showed a large variability in the performance of the EPM, ranging from lack of anxiety-like traits to enhanced behavioral responses to stimulus (Benno et al., 2009; Cai et al., 2017). In this study, metformin treatment during neonatal development had no anxiolytic effect in BTBR mice in the EPM or the light-dark tests, indicating that early metformin treatment reduces repetitive behaviors without inducing anxiety-like behaviors.

According to reports of the effects of metformin in rodents, the dose for metformin administration selected in our study, was equivalent to the dose (1,200 mg per 75 kg per day) used in humans, within the recommended dose range (<2,550 mg/day; Nair and Jacob, 2016; Tunc-Ozcan et al., 2018). We also found that there are no obvious effects on the body weight of the pups during the injection period, which suggests no observable side-effects on the pups. Considering that autism treatment should optimally be provided early in life, metformin administration from P7 to P14, closely approximates the developmental stage from the last trimester of human pregnancy, to the first few postnatal years in humans, which have a significant impact on neuroplasticity in the developing brain. Generally, there are no severe adverse events when metformin is used to treat pediatric populations (Jones et al., 2002). However, refinement of the dose and timing of metformin administration to young children, remains a concern.

The underlying mechanisms for ameliorating the behavioral phenotypes of ASD in BTBR mice by metformin administration, during early postnatal period, remains an interesting question. Signaling pathways interrupted by important cellular processes such as synaptic plasticity, neuromodulation and/or receptor trafficking or composition might be involved in the pathogenesis of ASD (Gilbert and Man, 2017; Oron and Elliott, 2017; Ohja et al., 2018). BTBR mice displayed abnormalities in mTOR signaling and inflammation signaling pathways, which can also be regulated by metformin (Onore et al., 2013; Steinmetz et al., 2018). Growing evidence has suggested that increased mTOR signaling pathways in the brain may play a critical role in the pathophysiology of ASD (Ehninger and Silva, 2011; Kaeberlein, 2013; Wang and Doering, 2013). Consistent with previous reports, our results indicated that both mTOR and S6K mRNA levels in the cerebral cortex of BTBR mice were significantly higher than that in B6 mice. Metformin treatment decreased mTOR gene expression in BTBR mice and showed a tendency to decrease in the S6K expression. In addition, metformin could significantly reduce the mTOR protein in a BTBR mouse cortex but not in a B6 mouse cortex. Autistic children showed abnormal immune responses along with the activation of the NF-κB signaling pathway (Nadeem et al., 2017). However, there was no statistical difference in the expression of NF-κB in the four groups. It is supposed that mTOR signaling might be involved in the behavioral alleviation in autism-like behaviors in BTBR mice mediated by metformin treatment.

In summary, this study discovered that metformin treatment during neonatal development, rescued the social deficits and reduced repetitive behavior in BTBR mice. The data presented in this study suggest that early intervention with metformin may provide a promising strategy to treat core symptoms of ASD. Metformin represents one potential pharmacological intervention.

## Author Contributions

LW participated in the experiments, data analysis and drafted the manuscript. YC participated in the experiments and data analysis. XF designed the study and revised the manuscript. All authors read and approved the final manuscript.

## Conflict of Interest Statement

The authors declare that the research was conducted in the absence of any commercial or financial relationships that could be construed as a potential conflict of interest.
